# Cost-effectiveness of strategies to control the spread of carbapenemase-producing Enterobacterales in hospitals: a modelling study

**DOI:** 10.1186/s13756-022-01149-0

**Published:** 2022-09-19

**Authors:** Lidia Kardaś-Słoma, Sandra Fournier, Jean-Claude Dupont, Lise Rochaix, Gabriel Birgand, Jean-Ralph Zahar, François-Xavier Lescure, Solen Kernéis, Isabelle Durand-Zaleski, Jean-Christophe Lucet

**Affiliations:** 1 INSERM, IAME, Université de Paris Cité, 75018 Paris, France; 2 AP-HP, Hôpital Bichat, URC, 75018 Paris, France; 3 AP-HP, Prévention du Risque Infectieux, Direction Patient Qualité Affaires Médicales, 75004 Paris, France; 4 Hospinnomics (PSE-AP-HP), Université de Paris, 75004 Paris, France; 5 Université de Paris 1 Panthéon-Sorbonne, Hospinnomics (PSE-AP-HP), 75004 Paris, France; 6 National Institute of Health Research Health Protection Research Unit in Healthcare Associated Infection and Antimicrobial Resistance, Imperial College London, London, UK; 7 Centre Hospitalo-Universitaire de Nantes, Nantes, France; 8 AP-HP, Hôpital Avicenne, Prévention du Risque Infectieux, GH Paris Seine Saint – Denis, 93000 Bobigny, France; 9 AP-HP, Hôpital Bichat, Maladies infectieuses et tropicales, 75018 Paris, France; 10 AP-HP, Hôpital Bichat, Equipe de Prévention du Risque Infectieux (EPRI), 75018 Paris, France; 11 CRESS, INSERM, INRA, URCEco, AP-HP, Hôpital de L’Hôtel Dieu, Université de Paris, 75004 Paris, France

**Keywords:** Cross-transmission, Carbapenemase-producing Enterobacterales, Hand disinfection, Mathematical model, Cost-effectiveness, Control strategies, France

## Abstract

**Background:**

Spread of resistant bacteria causes severe morbidity and mortality. Stringent control measures can be expensive and disrupt hospital organization. In the present study, we assessed the effectiveness and cost-effectiveness of control strategies to prevent the spread of Carbapenemase-producing Enterobacterales (CPE) in a general hospital ward (GW).

**Methods:**

A dynamic, stochastic model simulated the transmission of CPE by the hands of healthcare workers (HCWs) and the environment in a hypothetical 25-bed GW. Input parameters were based on published data; we assumed the prevalence at admission of 0.1%. 12 strategies were compared to the baseline (no control) and combined different prevention and control interventions: targeted or universal screening at admission (TS or US), contact precautions (CP), isolation in a single room, dedicated nursing staff (DNS) for carriers and weekly screening of contact patients (WSC). Time horizon was one year. Outcomes were the number of CPE acquisitions, costs, and incremental cost-effectiveness ratios (ICER). A hospital perspective was adopted to estimate costs, which included laboratory costs, single room, contact precautions, staff time, i.e. infection control nurse and/or dedicated nursing staff, and lost bed-days due to prolonged hospital stay of identified carriers. The model was calibrated on actual datasets. Sensitivity analyses were performed.

**Results:**

The baseline scenario resulted in 0.93 CPE acquisitions/1000 admissions and costs 32,050 €/1000 admissions. All control strategies increased costs and improved the outcome. The efficiency frontier was represented by: (1) TS with DNS at a 17,407 €/avoided CPE case, (2) TS + DNS + WSC at a 30,700 €/avoided CPE case and (3) US + DNS + WSC at 181,472 €/avoided CPE case. Other strategies were dominated. Sensitivity analyses showed that TS + CP might be cost-effective if CPE carriers are identified upon admission or if the cases have a short hospital stay. However, CP were effective only when high level of compliance with hand hygiene was obtained.

**Conclusions:**

Targeted screening at admission combined with DNS for identified CPE carriers with or without weekly screening were the most cost-effective options to limit the spread of CPE. These results support current recommendations from several high-income countries.

**Supplementary Information:**

The online version contains supplementary material available at 10.1186/s13756-022-01149-0.

## Introduction

Carbapenemase-producing Enterobacterales (CPE) are increasingly common in hospitals and represent a serious health problem. These multidrug-resistant organisms colonise the gastrointestinal tract after direct (person-to-person) or indirect (via contaminated surfaces) transmission. *Klebsiella Pneumoniae* and *Escherichia coli* are the common causes of urinary tract infections, ventilator-associated pneumonia and bloodstream infections in healthcare settings [1]. In 2015, in the EU/ EEA, the annual number of CPE infections was estimated as 15,947 for *K. Pneumoniae* and 2619 for *E. coli* [2]. Treatment options for patients infected with CPE are limited, leading to high mortality, and increased length of stay and hospital costs. The successful implementation of cost-effective infection control measures to prevent CPE spread and infections is key for hospital managers.

Recommendations to limit the transmission of CPE in healthcare facilities are based on the early detection of asymptomatic carriers, implementation of contact precautions and isolation in a single room [3–6]. In practice, strategies combining various interventions are employed according to the risk assessment and available resources: (1) universal or targeted rectal screening on admission, (2) standard precautions (SP), applied to all patients regardless of their infectious status, (3) contact precautions (CP) for identified carriers or infected patients, (4) isolation in a single room, (5) environmental cleaning, (6) rectal screening of contact patients, i.e. those whose care was provided by the same team as the CPE patient, and/or (7) isolation of carriers in a dedicated area with dedicated nursing staff (DNS), hereafter designated as cohorting.

However, these control measures pose challenges such as the high cost of patient screening and cohorting, requirement for single room isolation or staff shortage for implementing precautions. Moreover, the effectiveness and cost-effectiveness of various CPE control strategies is under-documented [4].

Mathematical models can be used to study the effectiveness, cost and cost-effectiveness of control strategies and to help decision-makers in the identification of the best combination of interventions to control the spread of antimicrobial-resistant organisms. These models make it possible to test the potential impact of different interventions before real-world implementation, which can save time and resources. Nevertheless, stringent strategies for controlling CPE have rarely been evaluated through mathematical modelling [7–10].

Our objective was to compare the impact, cost and cost-effectiveness of different strategies combining screening and contact precaution measures to control the spread of CPE in a general medicine ward (GW) using a mathematical model.

## Methods

### Model

**Fig. 1:**
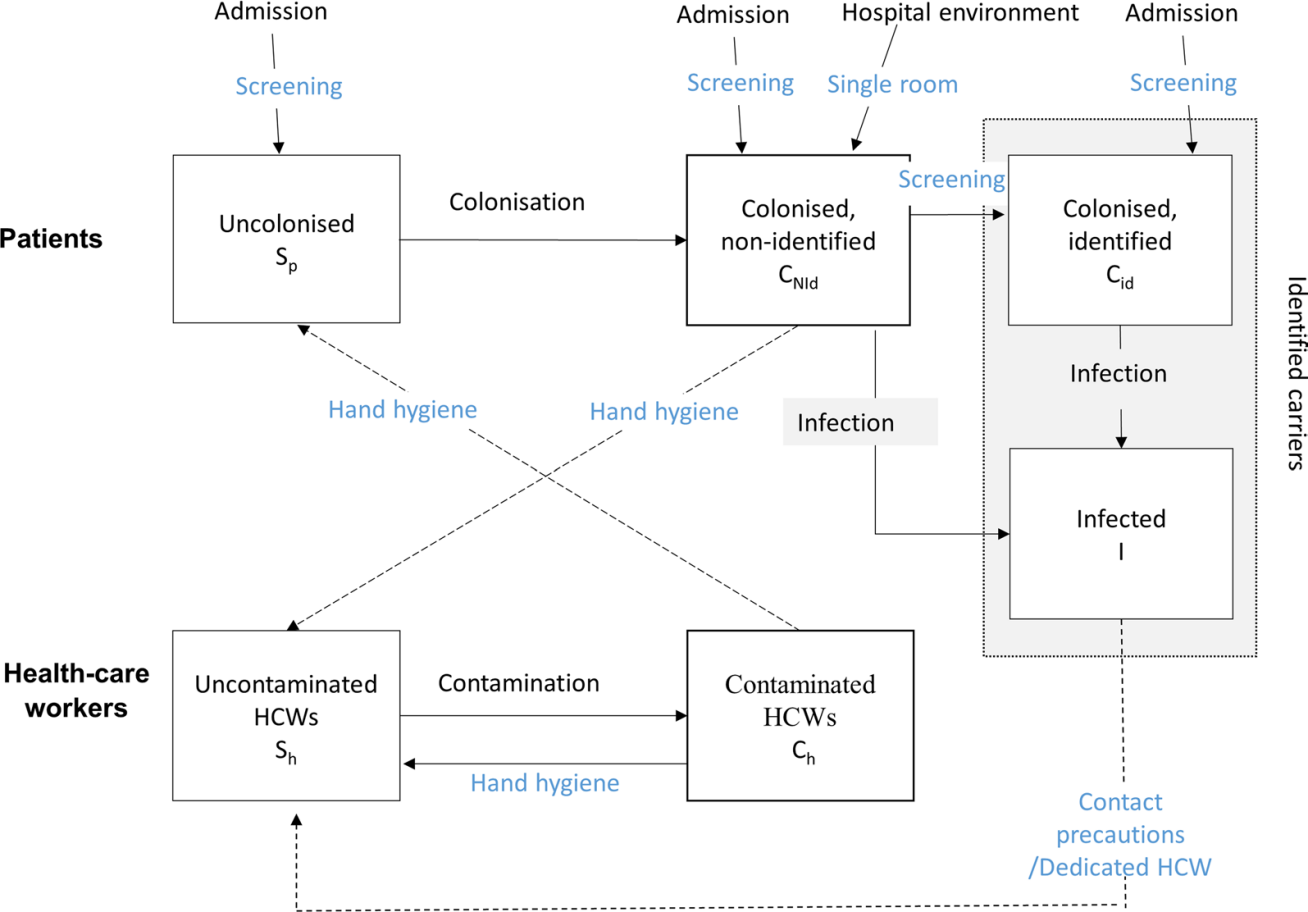
Flow diagram describing the CPE spread between patients and HCWs and the implementation of interventions. Possible interventions are indicated in blue: (1) hand hygiene, (2) contact precautions (better hand hygiene, gown and gloves), (3) dedicated staff (or cohorting of identified patients), (4) single room (limit the transmission by the environment), (5) screening on admission or during hospital stay

We used a compartmental, stochastic model [11, 12] to describe the dynamics of CPE transmission in a general medicine ward (GW). The model simulates hospital patient admission and discharge, the spread of a CPE between patients via contact with healthcare workers (HCWs) and the hospital environment. In this model, patients are classified as either uncolonised, colonised- unidentified, colonised- identified or infected. HCWs can be uncontaminated or transiently contaminated (hands) (Fig. 1). Additional file 1: Appendix A1 provides details of the model.

### Assumptions and parameters of model

We modelled a 25-bed GW, with the continuous presence of 10 HCWs (5 nurses and 5 nursing assistants) [13–15]. We assumed, for each patient each day, an average of 28 HCWs visits [13, 14]. Thus, the number of HCWs visits associated with at least one contact per HCW per day = 2.8.

At t0, we assumed a CPE-free ward and simulated the admission and discharge of patients. Bed occupancy was assumed to be 100%. The prevalence of CPE carriage at the time of hospital admission varies widely between countries but remains low in high-income Western-European countries [16, 17]. We assumed the baseline prevalence of CPE carriage on admission to be 0,1% [17–20]. Other scenarios for CPE carriage on admission were considered in a sensitivity analysis.

The model parameters are presented in Table 1. We used data from the literature and from university hospital trusts operating in Paris and its surroundings (AP-HP). Each day, each patient received an average of 28 HCW visits (or 2.8 visits per HCW per day) [13, 14]. During a contact with a colonised patient, an HCW could contaminate hands with CPE with a probability of b_h_ = 0.21 [7] and transmit bacteria to other patients. Uncolonised patients could become colonised (intestinal colonisation) after contact with a contaminated HCW. The unknown probability of intestinal colonisation (b_p_) was calibrated in order to reproduce an average number of secondary cases of 0.77 per patient (range between 0 and 3), observed in a multicentre study from three hospitals [21]. Our model was also calibrated on ESBL-PE from a large interventional European multicentre study, considering that CPE spread might match ESBL spread [22]. The parameter *b*_*p*_ obtained from this calibration was used in a sensitivity analysis.

**Table 1:** Input parameters and plausible ranges for sensitivity analysis

Parameter	Description	Value	Range	Source
N_p_	No. of beds	25	5–50	[13]
N_h_	Number of HCWs	10		[14, 15]
c_p_	Number of HCW visits associated with at least one aseptic contact per patient per day	28	21–39	[13, 14]
a	No. of HCW visits associated with at least one aseptic contact per HCW per day	2.8		c_p_/N_h_
**Mean length of stay (days)**				
d_S_	-Uncolonised	6.2	3.5–8	[47]
d_CNId_	-Colonised non-identified	12	1–50	[21]
d_I_	-Infected/colonised identified	25	4–150	[27, 29, 39, 48–50]
γ_S_	Discharge rate of uncolonised patients (per day)	0.16		1/d_S_
γ_CNId_	Discharge rate of colonised non-identified patients (per day)	0.08		1/ d_CNId_
γ_I_	Discharge rate of infected patients (per day)	0.04		1/ d_I_
b_p_	Colonisation probability for patients (/ contact)	0.021	0.009–0.021	Model’s calibration based on the literature[21, 22]
b_h_	Probability of contamination of an HCW with CPE during a contact with a colonised patient (/contact)	0.21	0.05–0.4	[7, 51–53]
μ_0_	Natural decontamination rate for HCW (i.e. not by hand hygiene) (/day)	24	12–48	[7, 54]
p_inf_	Probability of infection in colonised patient	0.0757	0.0541–0.1024	[9, 30, 39, 35, 55]
**Probability of death during hospital stay**				
pd_S_	-Uncolonised	0.020	0.012–0.036	[31]
pd_C_	-Colonised	0.036	0.018–0.054	[32]
pd_I_	-Infected	0.30	0.09–0.9	[9, 33, 39, 49, 56–58]
p_p_	Probability of hand hygiene before contact with patient (uncolonised or colonised unidentified)	0.4	0.06–0.9	[34]
p_h_	Probability of hand hygiene after contact with patient (uncolonised or colonised unidentified)	0.51	0.06–0.9	[34]
φ	Prevalence of CPE carriage among admitted patients	0.001	0.001–0.05	[17–20]
α	Colonisation rate by hospital environment (/day)	0.00001	0.00001–0.00041	[23–25]
**Control mesures’ specific parameters**				
r_a_	Part of colonised patients, identified at admission in a risk-based screening	0.5	0.2–0.5	Local data and [17, 35]
s_b_	Sensitivity of the screening method (%)	0.96	0.9–1	[59–61]
s_p_	Specificity of the screening method (%)	1	Assumed	
p_id_	Probability of hand hygiene before/after contact with identified CPE patient	0.8		[36, 62]
r_s_	Part of single room in the ward (%)	10	Assumed	

We assumed that uncolonised patients could also become colonised with CPE through the hospital environment at rate α = 0.00001 per day [23–25]. As CPE colonisation can persist for months [26], we assumed that patients who acquired CPE remained colonised during their hospital stay. The average length of stay (LOS) of uncolonised patients was set at 6 days. Colonisation and infection with CPE have been reportedly associated with increased hospital stay [27–29]. We assumed that LOS of infected or colonised-identified patients was 25 days, giving an excess LOS of 3 weeks in comparison with uncolonised patients as reported in other studies [27, 29]. The average LOS of colonised patients non-identified during hospital stay was estimated at 12 days [21]. In the model, the probability that a CPE-colonised person would develop symptomatic infection during hospital stay was 0.076 [30]. The probability of death during hospital stay for uncolonised, colonised and infected patients was 0.020, 0.036 and 0.3 respectively [31–33].

### Baseline scenario

In the baseline scenario, we assumed the following: standard contact precautions with 40% hand hygiene (HH) compliance before contact with a patient and 50% after patient contact [34], no rectal screening on admission and no additional contact measures.

### Infection control strategies

Twelve strategies were gradually implemented and compared to the baseline scenario.

The first six strategies combined targeted or universal screening at admission and control measures applied to identified CPE carriers:

1. Targeted screening (TS) + contact precautions (CP) without isolation of carriers in a single room,
2. TS + CP + isolation in a single room,
3. TS + dedicated nursing staff (DNS) + isolation of carriers in a single room,
4. Universal screening (US) + CP without isolation in a single room,
5. US + CP + isolation in a single room,
6. US + DNS + isolation in a single room.

Further strategies (7–12) consisted in the enforcement of previous control measures through the weekly screening of contact patients (WSC), that is, patients cared for by the same HCWs as the CPE-positive patient.

Targeted screening was defined as the screening of patients with a history of CPE infection or colonisation, patients with a history of foreign hospital stay within the last year and patients repatriated from a hospital abroad [4–6]. We estimated from the literature and from personal experience that current risk-based screening at hospital admission would identify 50% of CPE-positive patients [17, 35].

The compliance with HH before/after contact with a patient was 80%/80% in the strategy with contact precautions. This value corresponds to the upper bounds of the interval of HH compliance reported in several studies [34, 36].

In the strategy with the isolation of identified carriers in a single room, the colonisation rate by hospital environment was assumed to be 0.

For the strategy with dedicated nursing staff, we introduced the additional HCWs caring exclusively for identified patients. The number of dedicated HCWs depended on the number of identified patients; we assumed the ratio 1 dedicated HCW for max. 10 patients.

In the universal screening strategy, all admitted patients were screened with a rapid screening test (e.g. PCR) and a result obtained within 24 h. The positive PCR test had to be confirmed by culture, which was included in costs. Weekly screening was based on culture only. For simplicity, we assumed 100% specificity.

See the Additional file 1: Appendix A1 for more details on control strategies.

### Costs

The analysis was performed from a public hospital perspective. The cost of hospital day was estimated using the French severity-adjusted, diagnosis-related group. Other costs were drawn from the literature and from previous studies of our team [11, 18, 37]. Cost were expressed in 2021 Euro (1€ = 1.19$).

The cost of the baseline scenario (reference strategy) was considered to be the cost of HH at baseline level (cost of the alcohol-based hand rub and staffing time) and cost of extended stay for CPE infected patients.

The costs of control measures were split into: (1) cost of rectal screening (testing materials and laboratory costs) and culture for CPE confirmation, (2) cost of contact precautions (gown, gloves, improved HH, infection team staff time), (3) cost of single room isolation (if necessary, transformation of a double-room into a single-arranged room, with the resulting loss in revenue for the hospital due to “blocked beds” and reduced admissions), (4) cost of dedicated nursing staff, and (5) cost of extended stay of identified CPE carriers. See Additional file 1: Table S2 for more details and for cost parameters (Table 2).

**Table 2:** Results of cost-effectiveness analysis.

Strategy	Total cost/1000 admissions (€) (SD)	Increase** from the baseline (%)	Nb of CPE acquisitions/1000 admissions (SD)	Reduction** from the baseline (%)	Δ Cost/1000 admissions (€)	*Δ Nb of CPE acquisitions/1000 admissions*	*ICER (*€/avoided case)
*Baseline*	32,050 (2443)	–	0.93 (1.50)				
1. TS + CP	37,304 (5567)	16.4	0.78 (1.31)	16.5			Dominated*
2. TS + CP + single room	37,509 (5636)	17.0	0.68 (1.24)	26.6			Dominated*
8. TS + CP + single room + WSC	38,455 (6866)	20.0	0.66 (1.22)	28.8			Dominated*
7. TS + CP + WSC	38,560 (7285)	20.3	0.78 (1.32)	16.4			Dominated*
3. TS + DNS	42,320 (10,916)	32.0	0.33 (0.86)	63.9	10,270	0.59	17,407
9. TS + DNS + WSC	42,934 (11,641)	34.0	0.31 (0.79)	66.4	614	0.02	30,700
4. US + CP	86,165 (6716)	168.8	0.72 (1.26)	22.1			Dominated*
11.US + CP + single room + WSC	87,151 (7931)	171.9	0.62 (1.16)	33.0			Dominated*
10.US + CP + WSC	87,231 (8245)	172.2	0.72 (1.22)	22.2			Dominated*
5. US + CP + single room	87,345 (7204)	172.5	0.60 (1.17)	43.8			Dominated*
6. US + DNS	95,427 (13,446)	197.7	0.02 (0.19)	97.7			Dominated*
12.US + DNS + WSC	95,561 (13,553)	198.2	0.02 (0.18)	97.9	52,627	0.29	181, 472

*TS* targeted screening, *US* universal screening, *CP* contact precautions, *DNS* dedicated staff, *WS* weekly screening

^*^Dominated: a strategy is dominated it means that resulted in higher costs but less benefit, or had a higher ICER than that of a more effective

^**^The Increase/Reduction from the baseline is calculated as: |Strategy’s value—Baseline value|/Baseline value * 100

### Model simulations and outcomes

We ran the model over a 1-year period to capture all costs and health effects relevant to control strategies implemented.

Simulations of the model were performed using Gillespie’s method and programmed in C++ language. The outcomes (number of CPE acquisitions, cost of intervention and incremental cost-effectiveness ratio (ICER; the ratio of the difference in costs to difference in health benefits)) were calculated after a period of 1 year and as an average of 5000 Monte Carlo simulations. We calculated also the percentage increase/decrease in the costs and the number of CPE acquisitions from the baseline to each control strategy.

### Cost-effectiveness evaluation

The ICER between two strategies was defined as the additional cost of a specific strategy compared with the next least expensive strategy, divided by its additional clinical benefit (CPE acquisitions avoided). First, we sorted the strategies from the least to the most expensive. Then, we excluded the strategies that were dominated, it means that resulted in higher costs but less benefit, or had a higher ICER than that of a more effective alternative strategy. For the non-dominated strategies, we calculated the ICER and constructed an efficiency frontier comparing more costly, but more effective strategies.

### Sensitivity analysis

We performed several additional analyses to assess the impact of our assumptions and parameter uncertainty on the model’s outcomes.

### Deterministic sensitivity analysis

We first ran a univariate sensitivity analysis to consider the impact of a lower compliance with HH (60%/60%) in strategies with CP. Then we investigated the model with: (1) a reduced LOS of identified CPE cases (reduction by 50%), (2) a reduced LOS of one day for all categories of patients (that could be considered as home hospitalisation) and (3) with the LOS for unidentified CPE cases the same as for uncolonised patients (6 days). We also considered (1) a better identification of colonised patients in a risk-based screening at admission (90% vs 50% in central analysis), (2) higher prevalence of CPE carriage at admission (from 0.1%, to 1% or 5%) with less patients presenting with risk factors for CPE colonisation (20%). We then ran the model with a lower probability of colonisation, based on the another calibration [22].

We also performed a cost-effectiveness analysis with the cost of a hospital bed day higher than our baseline value (900 € vs 500€). We ran the model with a modified initial condition and assumed that a CPE identified carrier was present in a ward at t0 (in the central analysis we had a CPE-free ward at t0). Finally, we compared the effectiveness of standard precautions with the targeted screening + contact precautions for a different the level of HH compliance.

### Probabilistic sensitivity analysis

We performed a probabilistic sensitivity analysis to explore the effect of joint uncertainty across parameters (except strategy-specific parameters that were fixed) on the cost-effectiveness of strategies. In this analysis, we used triangular distributions for epidemiological or healthcare organisation parameters and gamma distributions for healthcare costs.

Then, we represented the cost-effectiveness acceptability curves graphically, showing the probability of each strategy having the highest net monetary benefit at different values of willingness to pay for a CPE case avoided.

## Results

For the strategy with standard contact precautions (baseline), over one year, 0.93 CPE acquisitions per 1000 admissions occurred. Among the cases, 92% were colonised from HCWs contaminated hands and 8% from the environment.

**Fig. 2:**
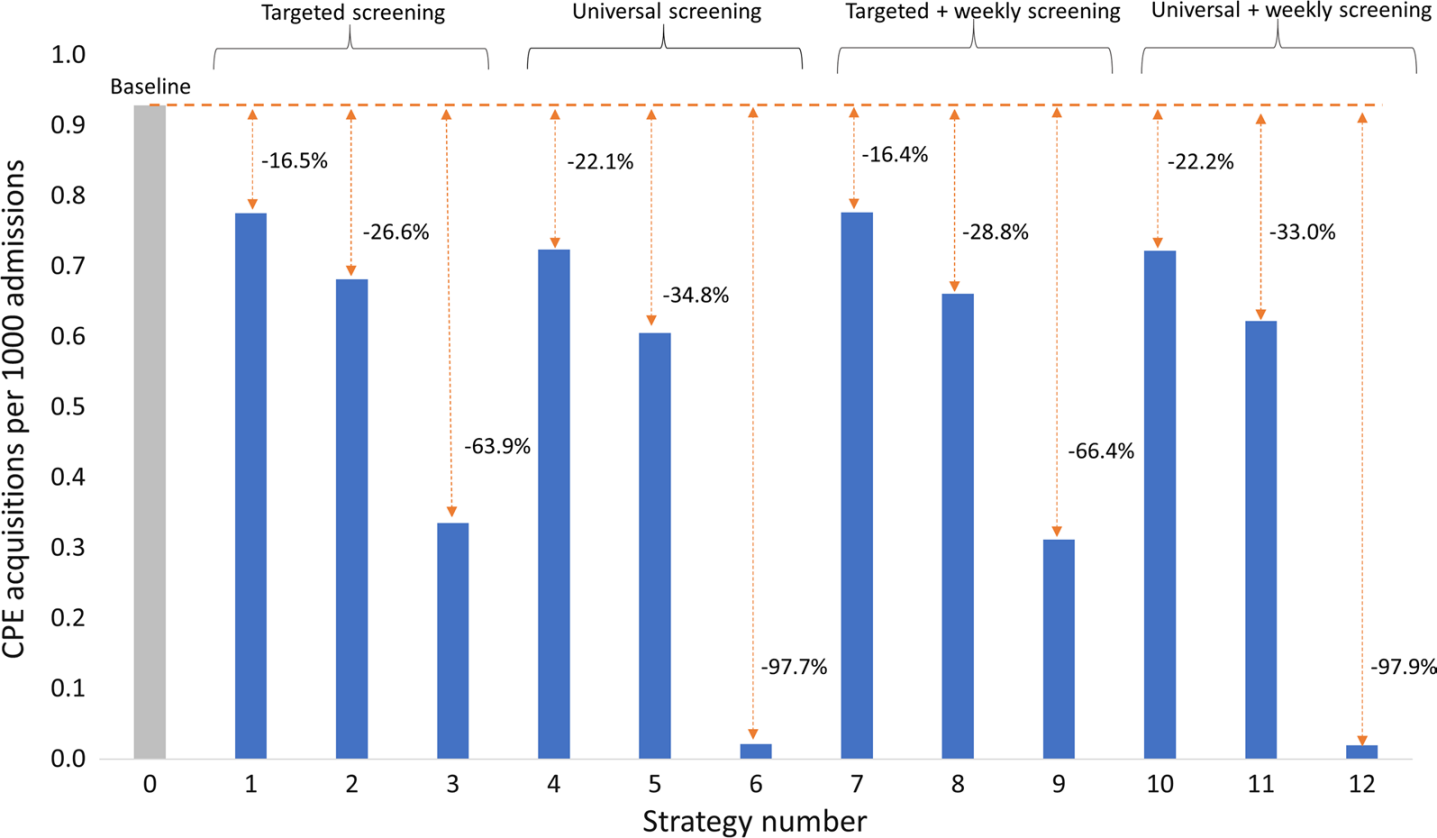
CPE acquisitions per 1000 admissions under different control strategies tested. Strategies: 0) standard contact precautions (baseline), (1) Targeted screening (TS) + contact precautions (CP) without isolation of carriers in single room, (2) TS + CP + single room, (3) TS + dedicated nursing staff (DNS) + single room, (4) Universal screening (US) + CP without isolation in single room, (5) US + CP + single room, (6) US + DNS + single room, (7) TS + CP + without isolation of carriers in single room + weekly screening of contact patients (WSC), (8) TS + CP + single room + WSC, (9) TS + DNS + single room + WSC, (10) US + CP without isolation in single room + WSC, (11) US + CP + single room + WSC, (12) US + DNS + single room + WSC

Compared to the baseline, all strategies were effective in limiting the spread of CPE (Fig. 2).

Strategies combining universal screening (US) with contact precautions (CP) or dedicated staff (DNS) reduced the number of cases by 22–98% and were more effective than those with targeted screening (TS) (reduction of 16–64%) (Table 2). Weekly screening (WSC) helped identify the additional number of CPE carriers but had little impact on nosocomial spread.

The most effective strategies combined screening on admission with dedicated staff for identified carriers. In strategy 3 (TS + DNS), we observed 0.34 CPE acquisitions / 1000 admissions (reduction of 64% compared to the baseline). In strategy 6 (US + DNS), there were 0.02 cases/1000 admissions (reduction of 98%). Strategies with weekly screening + DNS reduced CPE acquisition by 66% and 98% respectively.

Isolation of carriers in a single room + CP (strategies 2, 5, 8, 11) reduced the number of CPE acquisitions from 27 to 35% depending on the screening scenario chosen.

The least effective strategy combined screening with CP without single room isolation of identified carriers (strategies 1, 4, 7, 10). The reduction in cases ranged from 16 to 22% depending on the screening scenario.

### Cost-effectiveness of control strategies

The mean total cost of the baseline scenario was the lowest and estimated at €32,050/1000 admissions (Table 2).

Each control strategy led to health gains compared to the baseline but required higher resource utilization. The cost of strategies ranged from €37,304/1000 admissions to €95,561/1000 admissions with the most expensive strategies including US (strategies 4–6 and 10–12) (Table 2). In general, 84% to 90% of the cost was due to the implementation of control measures (additional personnel, screening, etc.) and 10% to 16% to the loss of hospital revenue due to the extended stay of identified carriers.

**Fig. 3:**
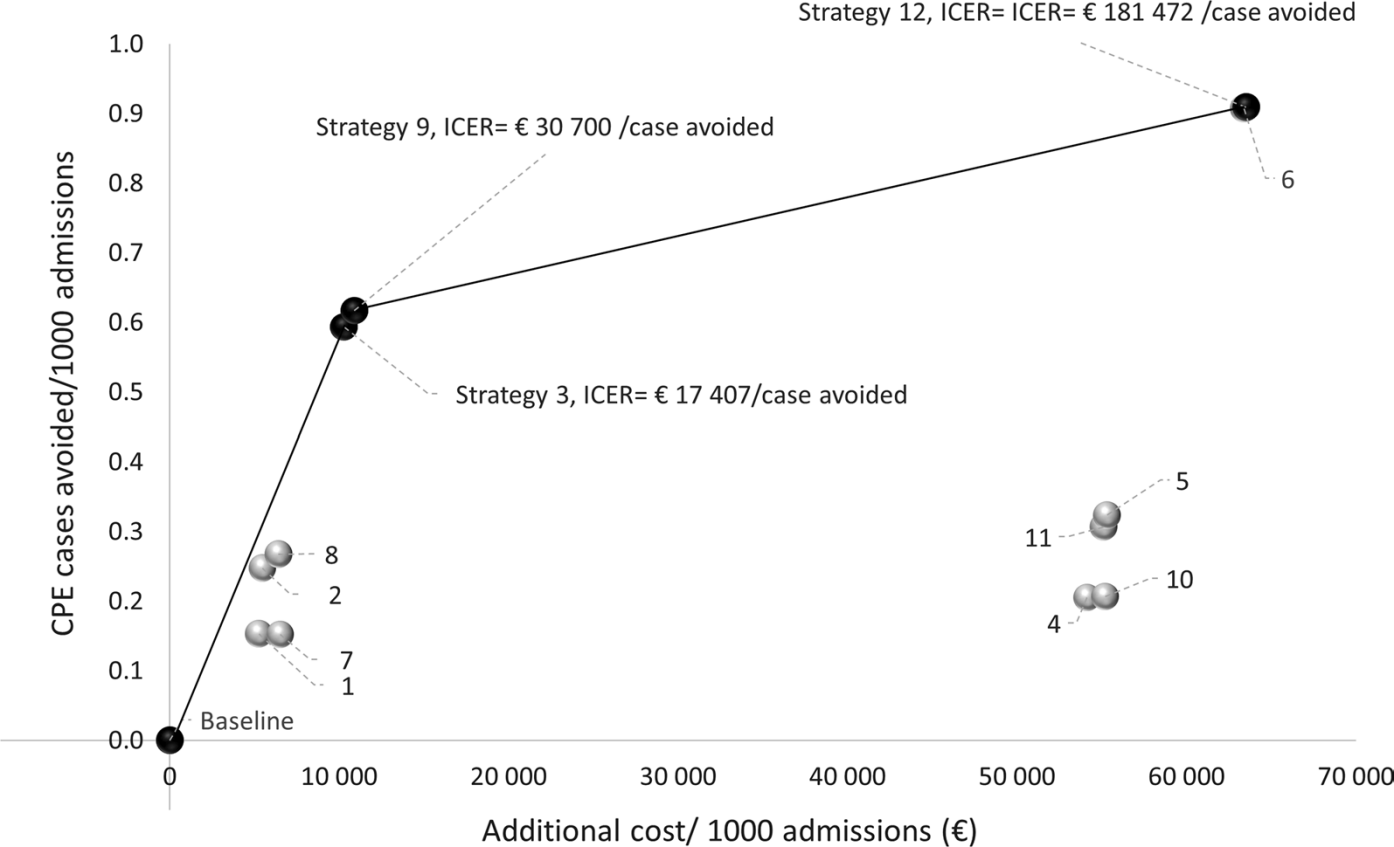
Cost-effectiveness plane showing the incremental benefits (CPE acquisitions avoided/1000 admissions) and costs relative to the least expensive strategy (baseline). Strategies (1, 2, 4, 5, 6, 7, 8, 10, 11) are dominated. The efficiency frontier (black line), joins the non-dominated strategies and the ICER between a specific strategy and the next, more costly, but more effective is presented

In the cost-effectiveness analysis, the efficiency frontier of prevention of CPE transmission was represented by strategies: 3) TS + dedicated nursing staff (DNS) + single room, 9) TS + DNS + single room + WSC, and 12) US + DNS + single room + WSC (Fig. 3). Other strategies were dominated.

The cost of moving from the base case to the strategy 3) TS + dedicated nursing staff (DNS) + single room was €17,407/ avoided CPE case. Moving from the strategy 3 to 9) TS + DNS + single room + WSC costs an additional €30,700/ avoided case. Finally, moving from the strategy 9 to 12) US + DNS + single room + WSC costs €181,472/avoided case.

### Deterministic sensitivity analysis

Findings from the sensitivity analysis confirmed the cost-effectiveness of strategies with dedicated staff for scenarios with: a reduced LOS of hospitalised patients, a lower probability of colonisation, a higher prevalence of CPE carriage at admission, a higher prevalence at admission combined with the lower identification of carriers by a risk-based screening, a CPE case identified at admission, and a higher cost of a hospital bed-day.

We have also found, that targeted screening combined with single room isolation of carriers and implementation of contact precautions were cost-effective if CPE carriers were identified upon admission or if the cases had a short stay in the ward. However, contact precautions were effective only when high level of compliance with HH was obtained. The summary, and details of results from these analyses, are presented in the Additional file 2: Appendix A2.

### Probabilistic sensitivity analysis

**Fig. 4:**
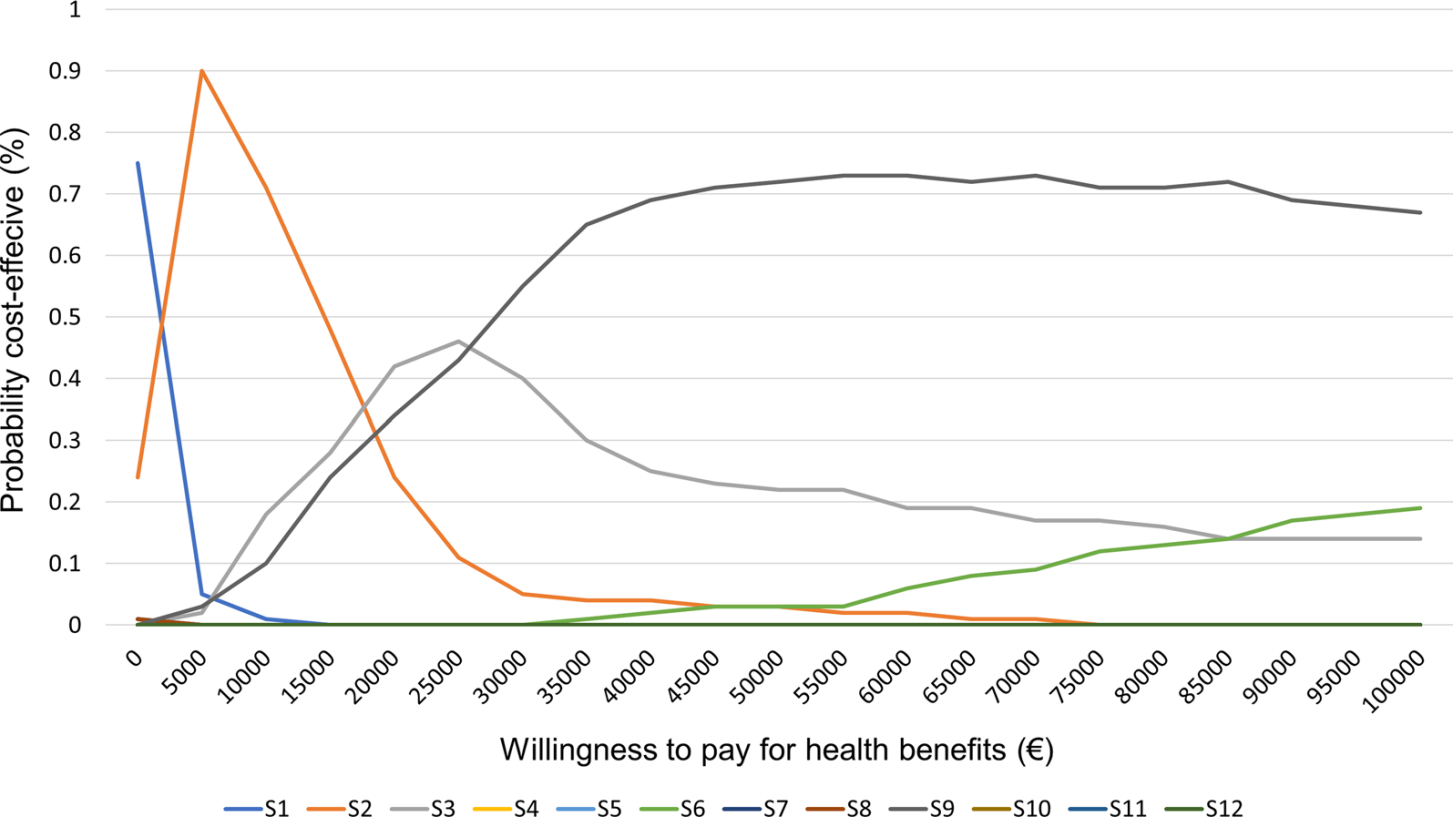
Cost-effectiveness acceptability curves for strategies. They represent the probability of each strategy to be cost-effective at different values of willingness to pay for a CPE case avoided. Strategies: (1) Targeted screening (TS) + contact precautions (CP) without isolation of carriers in single room, (2) TS + CP + single room, (3) TS + dedicated nursing staff (DNS) + single room, (4) Universal screening (US) + CP without isolation in single room, (5) US + CP + single room, (6) US + DNS + single room, (7) TS + CP + without isolation of carriers in single room + weekly screening of contact patients (WSC), (8) TS + CP + single room + WSC, (9) TS + DNS + single room + WSC, (10) US + CP without isolation in single room + WSC, (11) US + CP + single room + WSC, (12) US + DNS + single room + WSC

Results are presented as cost-effectiveness acceptability curves (Fig. 4), showing the probability of each strategy having the highest net monetary benefit at different values of willingness-to-pay threshold.

For example, at willingness-to-pay values lower than €675 per case avoided, strategy 1. TS + CP had the highest probability of being cost-effective (50–75%), followed by strategy 2. TS + CP + single room. At willingness-to-pay €17,000- 25,000, strategy 3. TS + DNS is an optimal option, in balance with strategy 9. TS + DNS + WSC and strategy 2. Finally, above €25,000 per case averted, strategy 9 became optimum.

## Discussion

The implementation of strategies to prevent the spread of CPE must take into consideration both costs and health benefits. In order to assist decision makers, we used a mathematical model of CPE transmission in a general medicine ward and evaluated the cost-effectiveness of twelve different strategies. We found that the targeted screening of at-risk patients at admission combined with dedicated staff for identified CPE carriers with or without weekly screening was the most cost-effective option.

Our findings show that in the baseline scenario, over one year, 0.93 CPE acquisitions per 1,000 admissions occurred. From an individual perspective, the likelihood of acquiring CPE (and infection) in a ward seems low. Nevertheless, from a national hospital perspective or from a societal perspective, the emergence of CPE remains worrying and can lead to outbreaks that are more difficult to manage, threaten patient’s safety, impact clinical services and are more costly to control than invested costs at the initial step of the epidemics [18, 27, 38].

We have shown that all strategies modelled were effective in reducing the number of CPE acquisitions. This reduction ranged from 16 to 98% depending on the intervention and was the greatest for strategies with dedicated staff (Table 2). Our results are consistent with other studies evaluating the effectiveness of control measures to limit the spread of highly resistant bacteria [39–41]. For example, Fournier et al. [39] have shown that the introduction of a control program in a large hospital network was very effective in reducing the number of secondary transmissions, despite the increasing number of CPE index cases. This study also underlined the importance of the early implementation of interventions after the identification of cases and the importance of dedicated staff.

Our results show that adding weekly screening had little impact on the nosocomial spread of CPE. This could be explained by the low prevalence of carriage at admission in our model. However, in real-life situations, where the cases are often discovered accidently during the hospital stay, weekly screening may help to identify additional CPEs and is important in the management of outbreaks.

In our study, the introduction of control strategies increased the cost by 16% to 34% (for strategies with targeted screening) and 169% to 198% (for strategies with universal screening) compared to baseline (Table 2). In terms of cost-effectiveness, the efficiency frontier of CPE prevention was represented by strategies combining targeted or universal screening at admission with dedicated staff. We also showed that the ICER increased steeply with universal screening. With a current prevalence of CPE carriage in Western-European countries (less than 1%), the willingness to pay for strategy 12 is rather hypothetical. The deterministic sensitivity analysis showed that if the prevalence is higher (e.g. 1–5%) or if the identification of carriers by a risk-based screening is weak, strategy 12 becomes more cost-effective (Additional file 2: Appendix A2, Table A6 and A7). These results are in accordance with another modelling study which found that universal screening could be cost-effective, depending on the prevalence of CPE colonization in admitted patients [9].

The implementation of a control strategy with dedicated staff (in a context of constrained budget and human resources) is a challenge for the hospital. According to data from Public Health France in 2019, dedicated staff were employed one out of one hundred times for the management of a CPE case discovered at admission and one out of ten times when a case was identified during the hospital stay [42]. Given the major impact of dedicated staff for controlling CPE spread, instituting cohorting of CPE carriers from different units within a single area appears desirable, at least in large hospitals affected by CPE. This measure was also included in the CPE management recommendations [4–6].

We investigated other hypotheses to validate the robustness of our predictions and to find conditions under which CP might be a cost-effective option. We showed that TS + CP + single room could be cost-effective when a CPE case was identified upon admission or when a case had a shorter stay in the ward (12 days instead of 25 days in the central analysis), but only if a high level of compliance with HH (80–80%) was obtained. Accelerating the transfer of CPE patients from acute care stay to dedicated rehabilitation units could be cost-effective.

Despite the recommendations [4–6] and the confirmed effectiveness of HH in prevention of nosocomial infections, compliance with HH remains low and often lower than the values used in our baseline scenario (40/50% before/after contact). In another analysis, we varied the level of HH and compared SP with the effectiveness of TS + CP. We found that a high HH compliance in SP might be even more effective than TS + CP (Additional file 2: Figure S1). This high level of compliance can be registered during audits of compliance with HH, but is actually rarely achieved when the compliance measurement is unobtrusive, with 2- to 2.5-fold lower compliance than through direct observation[43, 44]. However, improving compliance with HH must remain a central objective in controlling CPE spread, especially in hospitals where dedicated staff or cohorting is not an available option.

We also tested the realistic strategy of a higher prevalence of CPE carriage on admission (1%), with carriers presenting risk factors in only 20% of cases. Here again, the strategies with dedicated staff remained optimal, suggesting that these strategies should be used, even with an evolution towards an endemic CPE situation.

Our study has several strengths. First, we used a dynamic model to consider that the risk of colonisation depends on the number of carriers and can change over time. It also allows testing the effectiveness of interventions under different hypotheses (e.g. prevalence of CPE at admission). Second, our model was calibrated on various datasets, on CPEs [21] but also on ESBL-PE from a large European multicentre study [22], considering that CPE spread may not be different from ESBL spread. In addition, our study examined a range of control strategies combining various scenarios for both screening at admission as well as management of cases in the ward. Such an assessment using traditional epidemiological tools based on large, cluster-randomised studies is difficult, can be impacted by bias and does not make it possible to distinguish the individual impact of interventions.

In addition to the above points, our model has also taken into consideration the possibility that uncolonised patients could become colonised with CPE through the hospital environment. This way of transmission is rarely included in modelling studies. Finally, we tested the effectiveness and cost-effectiveness of strategies under different hypotheses and studied the impact of uncertainty in the estimation of the model’s parameters on our predictions. We also chose a hospital perspective which, while not recommended by international guidelines, is the most likely to convince hospital managers to the value of controlling CPE. While the healthcare system perspective considers all production costs, including those after hospital discharge, the hospital perspective looked at both cost and revenues during the hospital stay, which is what hospital managers do in systems driven by a Diagnosis-Related-Groups (DRG)-like prospective payment.

This analysis also allowed us to find the conditions under which the strategy based on CP was cost-effective, or to show that strategies with dedicated staff were the best options even if the prevalence of carriers at admission was higher.

Our study also has several limitations. First, we did not model the interruption of new admissions and transfers in an outbreak situation, as recommended by several guidelines. In our simulations, the situation of an outbreak was rare (occurring in about 4% of simulations), but we acknowledge that bed closures could represent the highest costs to contain an outbreak [18, 27].Second, the epidemiological characteristics of Enterobacterales are complex and may vary, depending on different species. For example, in the case of extended-spectrum beta-lactamase-producing Enterobacterales (ESBL-PE), several studies [22, 45] showed that ESBL *E. coli* was mainly imported and ESBL K. pneumoniae ESBL was mainly acquired. Furthermore, the differential capacity of cross-transmission between ESBL E. coli and other Enterobacterales has been clearly established [46]. We considered in our study that different enterobacterales carrying a carbepenemase or different carbapenemases conferred a similar impact for public health. We therefore did not differentiate these situations and decided to consider EPC globally.

Another potential limitation relates to a paucity of evidence about the length of stay for each patient category: either colonised-unidentified, identified or infected. In the literature, the additional length of stay of identified CPE cases (colonised identified/infected) compared to non-carrier patients was approximately 3 weeks [27, 29] and could be explained by the difficulties in transferring cases to downstream units. It is not clear whether the LOS of colonised-unidentified patients is longer than non-carriers. A multicentre study reported a LOS of unidentified colonised patients, incidentally discovered post-hospitalization, twice as long as the average LOS in the medical ward [21]. This extended LOS may be linked to other factors that promote CPE acquisition, e.g. intensity of care or exposure to antibiotics.

However, patient LOS is an important parameter for the model, with a strong impact on transmission and costs.

Finally, costs were estimated based on local and national data and may not be generalizable to other countries.

## Conclusions

In conclusion, our findings suggest that the targeted screening of at-risk patients at admission, combined with dedicated staff for identified CPE carriers with or without weekly screening, was the most cost-effective strategy to control the spread of CPE in a ward. This result holds true even though the prevalence of CPE carriage at admission is high and in variable sensitivity analyses. Targeted screening combined with isolation of carriers in a single room and implementation of contact precautions may merit consideration if CPE carriers are identified upon admission or if the cases have a short stay in the ward. However, contact precautions are effective only when a high level of compliance with HH is obtained.

## Supplementary Information

**Additional file 1**. Details of the model describing transmission dynamics of CPE in a hospital ward and control strategies.

**Additional file 2**. The summary and details of results from the deterministic sensitivity analysis.

## Data Availability

All data generated or analysed during this study are included in this article and its supplementary information files.

## Abbreviations

AP-HP: University hospital trusts operating in Paris and its surroundings
CP: Contact precautions
CPE: Carbapenemase-producing Enterobacterales
DNS: Dedicated nursing staff
GW: In a general hospital ward
HCWs: Healthcare workers
HH: Hand hygiene
ICER: Incremental cost-effectiveness ratio
LOS: Length of stay
TS: Targeted screening
US: Universal screening
WSC: Weekly screening of contact patients
