# Supplementary material for: Cost-effectiveness of strategies to control the spread of carbapenemase-producing Enterobacterales in hospitals: a modelling study
Source: Antimicrob Resist Infect Control. 2022 Sep 19;11:117. doi: 10.1186/s13756-022-01149-0 (PMC9484055; doi:10.1186/s13756-022-01149-0)
Supplement: Supplementary file 1 — Additional file 1. Details of the model describing transmission dynamics of CPE in a hospital ward and control strategies. [file 13756_2022_1149_MOESM1_ESM.docx]

# **Appendix A1**

| **Defining the objectives, scope and policy context of a model.** | |
| --- | --- |
| Decision objective | To evaluate CPE control strategies |
| Policy context | This analysis was used to support decision makers in choosing the best strategy for controlling CPE |
| Funding source | ARS-Ile-de-France*, PREPS Program** |
| Disease | CPE infections |
| Perspective | Hospital perspective |
| Target population | Patients hospitalised in a hypothetical general medicine ward |
| Health benefits | Reduction in CPE acquisitions |
| Strategies | Screening at admission (targeted or universal screening) combined with an infection control measures (strict contact precautions, single room, dedicated nursing staff, weekly screening of contact patients) |
| Resources/costs | Additional bed-days due to extended stay, laboratory/screening, IPC team time, dedicated staff, hh, gloves, gowns, isolation in single room |
| Time horizon | 1 year |
| * ARS-Ile de France- Health Agency, Paris Region  **PREPS - French government’s program on Care System Performance | |

**Transmission model**

The model describes transmission dynamics of CPE in a hospital ward and was adapted from that used in our previous work on the effectiveness and cost-effectiveness of ESBL-PE control strategies in an ICU[1,2]. The model was compartmental (population of patients and healthcare workers (HCWs) separated into mutually exclusives groups, based on their disease status) and stochastic (such that chance processes, important for small populations, are taken into account).

We simulated the contacts between patients and healthcare workers, the transmission of CPEs (through the contaminated hands of HCWs as well as the contaminated hospital environment), hospital admissions and discharges of patients and the different control strategies.

**Structure of the model in the absence of control measures (baseline scenario)**

In this model, at each time t, each patient was in one of the following states: uncolonised (S_p_), colonised- unidentified (asymptomatic carriers in the digestive tract) (C_NId_), or infected (symptomatic carriers) (I).

HCWs could be: uncontaminated (S_h_) or transiently contaminated (hands) (C_h_). The transmission of CPEs in the hospital was ensured by the nursing staff as well as the contaminated hospital environment. The model was simulated stochastically. A transition rate λ_i_ was attributed to each transition between states.

**Table 1** lists all transitions rates of our stochastic system. Simulations of the model were performed using Gillespie’s method [^1^](#_ENREF_1).

Table 1 Transition rates for stochastic compartmental model (baseline)

| Event | Transition | Rate |
| --- | --- | --- |
| Contamination of a HCW | (S_h_, C_h_) 🡪 (S_h_-1, C_h_+1) | λ_1_= β_h_ (C_NId_+ I) S_h_ |
| Decontamination of a HCW | (C_h_, S_h_) 🡪 (C_h_-1, S_h_+1) | λ_2_= µ C_h_ |
| Colonisation of a patient in contact with a HCWs | (S_p_, C_NId_)🡪 (S_p_-1, C_NId_ +1) | λ_3_=β_p_C_h_ S_p_ |
| Colonisation of a patient in contact with the environment | (S_p_, C_NId_)🡪 (S_p_-1, C_NId_ +1) | λ_4_= α S_p_ |
| Discharge and admission of an uncolonised patient | (S_p_, S_p_)🡪 (S_p_-1, S_p_+1) | λ_5_=γ_S_ S_p_ (1- φ) |
| Discharge of an uncolonised patient and admission of a colonised non-identified | (S_p_, C_NId_)🡪 (S_p_-1, C_NId_ + 1) | λ_5_= γ_S_ S_p_ φ |
| Discharge of a colonised, non-identified patient and admission of an uncolonised patient | (C_NId_, S_p_)🡪 (C_NId_ -1, S_p_+ 1) | λ_6_= γ_C_ C_NId_ ( 1- φ) |
| Discharge and admission of a colonised, non-identified patient | (C_NId_, C_NId_)🡪 (C_NId_-1, C_NId_ +1) | λ_7_= γ_C_ C_NId_ φ |
| Infection of a colonised patient | (C_NId,_ I) 🡪 (C_NId_-1, I+1) | λ_8 =_ C_NId_  p_inf_ |
| Discharge of an infected patient and admission of an uncolonised patient | (I, S_p_)🡪 (I- 1, S_p_+1) | λ_9_= γ_I_I (1- φ) |
| Discharge of an infected patient and admission of a colonised, non-identified patient | (I, C_NId_)🡪 (I- 1, C_NId_ +1) | λ_10_= γ_I_ I φ |
| Death of an uncolonised patient and admission of an uncolonised patient | (S_p_, S_p_)🡪 (S_p_-1, S_p_+1) | λ_11_= pd_S_ S_p_ (1- φ) |
| Death of an uncolonised patient and admission of a colonized, non-identified patient | (S_p_, C_NId_)🡪 (S_p_-1, C_NId_ + 1) | λ_12_= pd_S_ S_p_ φ |
| Death of a colonised patient and admission of uncolonised patient | (C_NId_, S_p_)🡪 (C_NId_ -1, S_p_+ 1) | λ_13_= pd_C_ C_NId_ ( 1- φ) |
| Death of a colonised patient and admission of a colonised patient | (C_NId_, C_NId_)🡪 (C_NId_-1, C_NId_ +1) | λ_14_= pd_C_ C_NId_ φ |
| Death of an infected patient and admission of an uncolonised patient | (I, S_p_)🡪 (I- 1, S_p_+1) | λ_15_= pd_I_  I (1- φ) |
| Death of an infected patient and admission of a colonised patient | (I, C_NId_)🡪 (I- 1, C_NId_ +1) | λ_16_= pd_I_ I φ |

Patients were admitted and discharged but bed occupancy was assumed to be 100% (the population of patient was constant).

Every day, HCWs were at risk of being contaminated by CPEs. The transmssion rate *β_h_* affects the transmission from the uncontaminated compartment of HCWs to the contaminated compartment in contact with a CPE colonised patient and was defined as follows:

*β_h_=* *a b_h_ (1- p_h_),*

where:

*a*: no. of HCW visits associated with at least one aseptic contact per HCW per day,

*b_h_* : probability of contamination of a HCW with CPE during a contact with a colonised patient,

*p_h_* : probability of hand hygiene after contact with patient

Uncolonised patient were at risk of being colonised by CPEs by contact with contaminated hospital environment (α) or by contaminated HCWs at rate *β_P_* defined as follows:

*β_P_ = a b_p_ (1- p_p_) ,*

where:

*a*: no. of HCW visits associated with at least one aseptic contact per HCW per day,

*b_p_*: probability of colonisation of a patient with CPE during a contact with a contaminated HCW,

*p_p_* : probability of hand hygiene before contact with patient.

Once colonised, patients do not clear CPEs colonisation before discharge. HCWs are transiently contaminated. The time to decontamination for a contaminated HCW follows an exponential distribution of mean 1/μ_0_ in the total absence of hand hygiene or of mean 1/μ (μ>μ_0_) in the presence of hand hygiene, with μ:

$$\mu=\mu_{0}+ a\left[ \left( 1-(1-p_{p})(1-p_{h}) \right) \left( S_{p}+C_{NId}(1-b_{h}) \right)+ p_{h}C_{NId}b_{h}+\left( 1-\left( 1-p_{id} \right)^{2} \right) I(1-b_{h})+ p_{id}Ib_{h} \right]$$

The second term on the right hand side of the equation (after “$\mu_{0}+$”) indicates that the time to HCWs’ hand decontamination depends on the rate of hand hygiene opportunities (*a*), on the probability that hand hygiene is completed (*p_p_* , *p_h, and_ p_id_* ) , and on the probability that transmission from a colonized patient to a HCW is successful (*b_h_*). To sum up, the hands of HCWs are decontaminated whenever HCWs perform hand hygiene:

- before or after contact with an uncolonized patients,
- before or after contact with a colonized patient that failed in contaminating them, and
- after contact with a contaminated patient who successfully contaminated them.

**Model calibration**

The model was simulated stochastically. We calibrated the colonisation parameter *b_p_* using Monte Carlo methods in order to reproduce the average number of secondary cases per patient observed in a French multicentre study after a 60-days period [3].

The parameter *b_p_* was also calibrated on ESBL-PE from a large European multicentre study testing standard vs contact precautions, considering that CPE spread may not be different from ESBL spread[4]. The parameter *b_p_* obtained from this calibration was used in a sensitivity analysis.

**Mathematical model with infection control strategies**

In order to study the impact of control strategies with screening scenario (on admission or a weekly screening), we added to the model one state for identified, CPE-colonised patients (C_Id_).

When strict contact precautions (CP) were applied, the transmission rate *β_HId_* described the contamination of a HCW from a CPE-identified patient and was defined as follows:

*β_HId_ = a b_h_ (1- p_Id_ ),*

where:

*a*: no. of HCW visits associated with at least one aseptic contact per HCW per day,

*b_h_* : probability of contamination of a HCW with CPE during a contact with a colonised patient,

*p_Id_* : probability of hand hygiene after contact with CPE-identified patient.

The transmission rates *β_h,_ β_P_*, and α remained the same to that of the baseline scenario .

In the strategy with the isolation of identified carriers in a single room, the colonisation rate by hospital environment was assumed to be 0 (α_Id_=0).

Finally, in the strategy with dedicated staff, a dedicated HCW visited only identified, colonised patients. Therefore, in the model considered, there was no cross-transmission from identified carriers to other patients, neither by HCWs nor the environment (*β_HId_ = 0*, α_Id_= 0). The transmission rates *β_h,_ β_P_*, and α remained the same to that of the baseline scenario for non-identified patients.

**Model simulations and outcomes**

Simulations of the model were performed using Gillespie’s method and programmed in C++ language. The outcomes were calculated after a period of 1 year and averaged over the 5,000 Monte Carlo simulations. Cost-effectiveness analysis were performed in R[5].

**Costs of control strategies**

The analysis was performed from a public hospital perspective. We estimated the costs of control strategies over the one-year simulation period. See Table 2 for details on cost parameters.

**The cost of the baseline scenario** (reference strategy) was considered to be the cost of HH at baseline level (cost of the alcohol-based hand rub and staffing time).

**The cost of screening** was first based on the cost of testing materials and on the cost of laboratory technician time spend on a rapid screening test (e.g. PCR). If PCR positive, we calculated the cost of performing cultures for CPE.

For the strategy in which **strict contact precautions** (CP) were implemented, we included the cost of improved HH, gowns and gloves (material and time of HCWs) and the supplementary time of an infection control nurse and an epidemiologist working on the management of CPE cases.

The cost of **isolation of CPE cases in single room** were calculated according to the number of patients needing isolation (p_is_) and the number of single rooms available in the ward (b_a_). We assumed that 10% of rooms in the ward were single rooms, available for patients’ isolation. If p_is_ > b_a_, we calculated the cost of transformation of a double-room into a single-arranged room, with the resulting loss in revenue for the hospital due to blocked beds and reduced admissions.

For the strategy where, **dedicated staff** were implemented, we calculated the cost of isolation in a single room and additional HCWs (one nurse and nursing assistant for max. 10 patients in isolation). To assure the continues presence of dedicated staff, we multiplied the number of dedicated staff by 3 (daily shifts).

The cost of **weekly screening** of contact patients was calculated as the cost of performing cultures for CPE.

Finally, we considered that extended length of stay of colonised and infected patients lead to the impossibility of taking care of new patients and weighted on hospital’s revenues.

The cost of lost bed days related to the extended length of stay of CPE patients was calculated for each strategy and based on the mean cost of hospital-day.

Table 2 Cost parameters, their sources and reported ranges.

| Cost Category | Value (€) | | Range | Source | |  |  |
| --- | --- | --- | --- | --- | --- | --- | --- |
| **Screening at admission** |  | |  |  | |  |  |
| Test PCR | 27 | | 20-34 | [6] | |  |  |
| Staff time to perform the test, complete the documentation etc. (15 min) | 5.9^a^ | | 5.1- 8.1 | [7,8] | |  |  |
| Negative culture | 0.7 | |  | [9] | |  |  |
| Positive culture | 115 | |  | [9] | |  |  |
| **Contact precautions** |  | |  |  | |  |  |
| HH (time: 30 sec. and alcohol-based hand rub: 3ml/ hand disinfection) | 0.203^a^ | | 0.124- 0.295 | [8,10] | |  |  |
| Infectious prevention and control (IPC) staff (10 min/identified pat-day) | 4.8 ^a^ | | 4.3 – 5.4 | [7,8] | |  |  |
| IPC meetings: IPC nurse + hospital epidemiologist (30 min/week) | 48 ^a^ | | 40.3-57.8 | [7,8] | |  |  |
| Gloves + gowns (material and time: 1min/ contact) | 0.95^a^ | | 0.9- 1.10 | [9] | |  |  |
| **CP+ single room** |  | |  |  | |  |  |
| CP | CP’s elements shown above | | | | |  |  |
| Mean cost of hospital-day | 500 | | 500-900 | [11] | |  |  |
| **Dedicated staff** |  | |  |  | |  |  |
| Nurse /day | 111 ^a^ | | 94.4-151.8 | [8] | |  |  |
| Nursing assistant /day | 89 ^a^ | | 80.4-114.5 | [8] | |  |  |
| CP | CP’s elements shown above | | | | |  |  |
| Mean cost of hospital-day | 500 | 500-900 | | | [11] |  |  |
| **Weekly screening** |  | |  |  | |  |  |
| Positive culture | 115 | |  | [9] | |  |  |
| **Additional bed-days due to extended**  **length of stay for case patients** |  | |  |  | |  |  |
| Mean cost of hospital-day | 500 | | 500-900 | [11] | |  |  |

*Cost of staff from a hospital perspective (salary + employer contributions).

**References**

1. Pelat C, Kardaś-Słoma L, Birgand G, Ruppé E, Schwarzinger M, Andremont A, et al. Hand Hygiene, Cohorting, or Antibiotic Restriction to Control Outbreaks of Multidrug-Resistant Enterobacteriaceae. Infect Control Hosp Epidemiol. 2016;37:272–80.

2. Kardaś-Słoma L, Lucet J-C, Perozziello A, Pelat C, Birgand G, Ruppé E, et al. Universal or targeted approach to prevent the transmission of extended-spectrum beta-lactamase-producing Enterobacteriaceae in intensive care units: a cost-effectiveness analysis. BMJ Open. 2017;7:e017402.

3. Saliba R, Neulier C, Seytre D, Fiacre A, Faibis F, Leduc P, et al. Can real-time polymerase chain reaction allow a faster recovery of hospital activity in cases of an incidental discovery of carbapenemase-producing Enterobacteriaceae and vancomycin-resistant Enterococci carriers? Journal of Hospital Infection. 2019;103:115–20.

4. Maechler F, Schwab F, Hansen S, Fankhauser C, Harbarth S, Huttner BD, et al. Contact isolation versus standard precautions to decrease acquisition of extended-spectrum β-lactamase-producing Enterobacterales in non-critical care wards: a cluster-randomised crossover trial. The Lancet Infectious Diseases. 2020;20:575–84.

5. R Core Team. R: The R Project for Statistical Computing [Internet]. 2016 [cited 2016 Sep 15]. Available from: https://www.r-project.org/

6. Birgand G, Ruimy R, Schwarzinger M, Lolom I, Bendjelloul G, Houhou N, et al. Rapid detection of glycopeptide-resistant enterococci: impact on decision-making and costs. Antimicrob Resist Infect Control. 2013;2:30.

7. Otter JA, Burgess P, Davies F, Mookerjee S, Singleton J, Gilchrist M, et al. Counting the cost of an outbreak of carbapenemase-producing Enterobacteriaceae: an economic evaluation from a hospital perspective. Clin Microbiol Infect. 2017;23:188–96.

8. Emploi-Collectivités. Grille indiciaire hospitalière : infirmier en soins généraux (ide) tout grade - fph [Internet]. Emploi-collectivités. 2021 [cited 2021 Apr 6]. Available from: https://www.emploi-collectivites.fr/grille-indiciaire-hospitaliere-infirmier-soins-generaux-ide/1/183.htm

9. Kardaś-Słoma L, Lucet J-C, Perozziello A, Pelat C, Birgand G, Ruppé E, et al. Universal or targeted approach to prevent the transmission of extended-spectrum beta-lactamase-producing Enterobacteriaceae in intensive care units: a cost-effectiveness analysis. BMJ Open. 2017;7:e017402.

10. Girard R, Aupee M, Erb M, Bettinger A, Jouve A. Hand rub dose needed for a single disinfection varies according to product: a bias in benchmarking using indirect hand hygiene indicator. J Epidemiol Glob Health. 2012;2:193–8.

11. Assistance publique – Hôpitaux de Paris (AP-HP). Base de données PMSI. 2015.
