# Supplementary material for: Cost-effectiveness of strategies to control the spread of carbapenemase-producing Enterobacterales in hospitals: a modelling study
Source: Antimicrob Resist Infect Control. 2022 Sep 19;11:117. doi: 10.1186/s13756-022-01149-0 (PMC9484055; doi:10.1186/s13756-022-01149-0)
Supplement: Supplementary file 2 — Additional file 2. The summary and details of results from the deterministic sensitivity analysis. [file 13756_2022_1149_MOESM2_ESM.docx]

# **Appendix A2**

**Summary of results from the deterministic sensitivity analysis.**

| **Central analysis** | **Summary of results** |
| --- | --- |
| Control strategies compared to the baseline | The efficiency frontier is given by the strategies:  3. Targeted screening + dedicated staff (€17,310 / avoided case),  9. Targeted screening + dedicated staff + weekly screening (€26,322/ avoided case),   1. Universal screening + dedicated staff + weekly screening (€179,839/avoided case). |
| **Scenario tested in the sensitivity analysis** | **Summary of results** |
| 1. Screening at admission + low compliance with HH in strategies with CP (60% vs 80% in central analysis) | - Increase in CPE transmission up to 56.9% due to the longer stay of identified carriers and decreased HH. |
| 2. Reduced LOS of CPE-identified carriers (12 days vs 25) | - Strategies with contact precautions become more effective in reducing CPE cases.  - Strategies with dedicated staff are still the most effective.  - Overall reduction in the cost of strategies.  - Strategies combining targeted screening with contact precautions+ single room are now on the efficiency frontier. |
| 3. One-day reduction in the LOS of all patients | - Decrease in the number of CPE cases in the baseline scenario with PS.  - Improved effectiveness of strategies with contact precautions.  - No impact on the effectiveness of the strategies with dedicated staff, which remained the most effective and cost effective. |
| 4. Better identification of carriers by a risk-based screening at admission (90% vs 50% in central analysis) | -Higher cost of strategies with targeted screening (by 10-15%) but improved effectiveness. |
| 5. Lower probability of colonisation (b_p_) (0,009 vs 0,021 in central analysis) | - Increased effectiveness of screening strategies with isolation of carriers in a single room.  - Strategies with dedicated staff remained the most effective. |
| 6. Higher prevalence of CPE carriage at admission (5% vs 0,01% in central analysis) | - Number of nosocomial cases increased.  - Little impact on strategies with dedicated staff remaining the most effective. |
| 7. Higher prevalence at admission combined with the lower identification of carriers by a risk-based screening (possible conditions in a few years) | - Lower effectiveness of strategies with targeted screening.  - Confirmed cost-effectiveness of strategies with dedicated staff. |
| 8. Identified CPE case present at model initiation (vs CPE free ward in central analysis) | - Effectiveness of all control strategies increases, the most so for strategies with contact precautions.  - Strategy of the targeted screening + contact precautions + single room becomes cost-effective. |
| 9. Higher cost of a hospital bed-day (1 000 € vs 500 €) | - Cost of strategies increased by 10-16%.  - Robustness of cost-effectiveness results for strategies with dedicated staff. |
| 10. LOS of unidentified CPE cases = LOS of uncolonisad patients | - Confirmed cost-effectiveness of strategy with targeted screening+ dedicated nursing staff |
| 11. Level of HH in SP varied from 40-80% | - Improve the HH compliance in standard precautions might be even more efficient than targeted screening + contact precautions |

**Results of deterministic sensitivity analysis**

**Table 1. Impact of lower HH compliance in strategies with strict contact precautions (CP) (60% vs 80% in central analysis).**

| Strategy | Total cost/1000 admissions | Nb of CPE acquisitions/1000 admissions | Nb of CPE acquisitions/1000 admissions (central analysis) | Δ Cost/1000 admissions (€) | Δ Nb of CPE acquisitions/1000 admissions | ICER (€) |
| --- | --- | --- | --- | --- | --- | --- |
| Baseline | 32,050 | 0.929 | 0.929 |  |  |  |
| 1. TS + CP | 37,513 | 1.113 | 0.776 |  |  | Dominated |
| 2. TS + CP + single room | 37,591 | 1.013 | 0.681 |  |  | Dominated |
| 7. TS+ CP + WSC | 39,894 | 1.211 | 0.777 |  |  | Dominated |
| 8. TS+ CP + single room + WSC | 39,914 | 1.109 | 0.661 |  |  | Dominated |
| 3. TS + DNS | 42,320 | 0.335 | 0.335 | 10,270 | 0.59 | 17,407 |
| 9. TS + DNS + WSC | 42,934 | 0.312 | 0.312 | 614 | 0.02 | 30,700 |
| 4. US + CP | 86,405 | 1.309 | 0.724 |  |  | Dominated |
| 5. US + CP + single room | 87,641 | 1.249 | 0.605 |  |  | Dominated |
| 10. US + CP+ WSC | 89,304 | 1.458 | 0.722 |  |  | Dominated |
| 11. US + CP + single room + WSC | 89,499 | 1.364 | 0.622 |  |  | Dominated |
| 6. US + DNS | 95,427 | 0.022 | 0.022 |  |  | Dominated |
| 12. US + DNS + WSC | 95,561 | 0.019 | 0.019 | 52,627 | 0.29 | 181,472 |

TS- targeted screening, US- universal screening, WSC- weekly screening

**Table 2. Impact of reduction in the LOS of CPE-identified carriers (12 vs 25 days in central analysis).**

| Strategy | Total cost/1000 admissions | Nb of CPE acquisitions/1000 admissions | | Nb of CPE acquisitions/1000 admissions (central analysis) | | Δ Cost/1000 admissions (€) | Δ Nb of CPE acquisitions/1000 admissions | ICER (€) |
| --- | --- | --- | --- | --- | --- | --- | --- | --- |
| Baseline | 32,050 | 0.929 | 0.929 | |  | |  |  |
| 2. TS + CP + single room | 32,737 | 0.529 | 0.681 | | 687 | | 0.40 | 1,718 |
| 1. TS + CP | 32,783 | 0.636 | 0.776 | |  | |  | Dominated |
| 8. TS+ CP + single room + WSC | 32,800 | 0.519 | 0.661 | | 63 | | 0.01 | 6,300 |
| 7. TS+ CP + WSC | 32,841 | 0.628 | 0.777 | |  | |  | Dominated |
| 3. TS + DNS | 35,837 | 0.328 | 0.335 | | 3,100 | | 0.19 | 16,316 |
| 9. TS + DNS + WSC | 35,944 | 0.314 | 0.312 | |  | |  | Dominated |
| 4. US + CP | 77,147 | 0.434 | 0.724 | |  | |  | Dominated |
| 11. US + CP + single room + WSC | 77,238 | 0.340 | 0.622 | |  | |  | Dominated |
| 10. US + CP+ WSC | 77,250 | 0.432 | 0.722 | |  | |  | Dominated |
| 5. US + CP + single room | 77,676 | 0.358 | 0.605 | |  | |  | Dominated |
| 12. US + DNS + WSC | 82,240 | 0.025 | 0.019 | | 46,403 | | 0.30 | 154,677 |
| 6. US + DNS | 82,252 | 0.027 | 0.022 | |  | |  | Dominated |

TS- targeted screening, US- universal screening, WSC- weekly screening.

**Table 3. Impact of one-day reduction in the LOS of all patients.**

| Strategy | Total cost/1000 admissions | Nb of CPE acquisitions/1000 admissions | Nb of CPE acquisitions/1000 admissions (central analysis) | Δ Cost/1000 admissions (€) | Δ Nb of CPE acquisitions/1000 admissions | ICER (€) |
| --- | --- | --- | --- | --- | --- | --- |
| Baseline with reduced LOS | 26,819 | 0.81 |  |  |  | - |
| 2. TS + CP + single room | 31,709 | 0.59 | 0.681 |  |  | Dominated |
| 1. TS + CP | 31,730 | 0.66 | 0.776 |  |  | Dominated |
| Baseline | 32,050 | 0.93 | 0.929 |  |  | Dominated |
| 7. TS+ CP + WSC | 32,591 | 0.65 | 0.777 |  |  | Dominated |
| 8. TS+ CP + single room + WSC | 32,614 | 0.59 | 0.661 |  |  | Dominated |
| 3. TS + DNS | 36,253 | 0.29 | 0.335 |  |  | Dominated |
| 9. TS + DNS + WSC | 36,778 | 0.25 | 0.312 | 9,959 | 0.56 | 17,784 |
| 4. US + CP | 80,446 | 0.62 | 0.724 |  |  | Dominated |
| 11. US + CP + single room + WSC | 81,070 | 0.54 | 0.622 |  |  | Dominated |
| 10. US + CP+ WSC | 81,206 | 0.61 | 0.722 |  |  | Dominated |
| 5. US + CP + single room | 81,369 | 0.56 | 0.605 |  |  | Dominated |
| 12. US + DNS + WSC | 88,125 | 0.02 | 0.019 | 51,347 | 0.23 | 223,248 |
| 6. US + DNS | 88,174 | 0.03 | 0.022 |  |  | Dominated |

TS- targeted screening, US- universal screening, WSC- weekly screening

**Table 4. Impact of better identification of carriers by a risk-based screening at admission (90% vs 50% in central analysis).**

| Strategy | Total cost/1000 admissions | Nb of CPE acquisitions/1000 admissions | Nb of CPE acquisitions/1000 admissions (central analysis) | Δ Cost/1000 admissions (€) | Δ Nb of CPE acquisitions/1000 admissions | ICER (€) |
| --- | --- | --- | --- | --- | --- | --- |
| Baseline | 32,050 | 0.929 | 0.929 |  |  |  |
| 1. TS + CP | 41,045 | 0.761 | 0.776 |  |  | Dominated |
| 2. TS + CP + single room | 41,251 | 0.623 | 0.681 |  |  | Dominated |
| 7. TS+ CP + WSC | 41,906 | 0.744 | 0.777 |  |  | Dominated |
| 8. TS+ CP + single room + WSC | 41,942 | 0.604 | 0.661 |  |  | Dominated |
| 3. TS + DNS | 49,134 | 0.088 | 0.335 |  |  | Dominated |
| 9. TS + DNS + WSC | 49,246 | 0.080 | 0.312 | 17,196 | 0.85 | 20,231 |
| 4. US + CP | 86,165 | 0.724 | 0.724 |  |  | Dominated |
| 11. US + CP + single room + WSC | 87,151 | 0.622 | 0.622 |  |  | Dominated |
| 10. US + CP+ WSC | 87,231 | 0.722 | 0.722 |  |  | Dominated |
| 5. US + CP + single room | 87,345 | 0.605 | 0.605 |  |  | Dominated |
| 6. US + DNS | 95,427 | 0.022 | 0.022 |  |  | Dominated |
| 12. US + DNS + WSC | 95,561 | 0.019 | 0.019 | 46,314 | 0.06 | 771,900 |

TS- targeted screening, US- universal screening, WSC- weekly screening

**Table 5. Impact of lower probability of colonisation (b_p_) (0.009 vs 0.021 in central analysis).**

| Strategy | Total cost/1000 admissions | Nb of CPE acquisitions/1000 admissions | Nb of CPE acquisitions/1000 admissions (central analysis) | Δ Cost/1000 admissions (€) | Δ Nb of CPE acquisitions/1000 admissions | ICER (€) |
| --- | --- | --- | --- | --- | --- | --- |
| Baseline | 31,806 | 0.346 | 0.929 |  |  |  |
| 1. TS + CP | 37,149 | 0.292 | 0.776 |  |  | Dominated |
| 2. TS + CP + single room | 37,200 | 0.232 | 0.681 |  |  | Dominated |
| 8. TS+ CP + single room + WSC | 37,741 | 0.219 | 0.661 |  |  | Dominated |
| 7. TS + PCC +WSC | 37,757 | 0.281 | 0.777 |  |  | Dominated |
| 3. TS + DNS | 42,097 | 0.113 | 0.335 | 10,291 | 0.23 | 44,743 |
| 9. TS + DNS + WSC | 42,634 | 0.116 | 0.312 |  |  | Dominated |
| 4. US + CP | 85,950 | 0.270 | 0.724 |  |  | Dominated |
| 10. US + CP+ WSC | 86,197 | 0.262 | 0.722 |  |  | Dominated |
| 11. US + CP + single room + WSC | 86,475 | 0.206 | 0.622 |  |  | Dominated |
| 5. US + CP + single room | 87,088 | 0.202 | 0.605 |  |  | Dominated |
| 6. US + DNS | 95,319 | 0.008 | 0.022 |  |  | Dominated |
| 12. US + DNS + WSC | 95,559 | 0.007 | 0.019 | 53,463 | 0.11 | 486,027 |

TS- targeted screening, US- universal screening, WSC- weekly screening.

**Table 6. Impact of higher prevalence of CPE carriage at admission (5% vs 0.01% in central analysis).**

| Strategy | Total cost/1000 admissions | Nb of CPE acquisitions/1000 admissions | Nb of CPE acquisitions/1000 admissions (central analysis) | Δ Cost/1000 admissions (€) | Δ Nb of CPE acquisitions/1000 admissions | ICER (€) |
| --- | --- | --- | --- | --- | --- | --- |
| Baseline | 67,050 | 25.89 | 0.929 |  |  |  |
| 1. TS + CP | 250,215 | 19.94 | 0.776 |  |  | Dominated |
| 2. TS + CP + single room | 252,246 | 19.91 | 0.681 |  |  | Dominated |
| 3. TS + DNS | 328,815 | 10.59 | 0.335 | 261,765 | 15.30 | 17,109 |
| 7. TS+ CP + WSC | 466,239 | 16.83 | 0.777 |  |  | Dominated |
| 4. US + CP | 466,900 | 17.01 | 0.724 |  |  | Dominated |
| 10. US + CP+ WSC | 555,893 | 15.83 | 0.722 |  |  | Dominated |
| 9. TS + DNS + WSC | 562,225 | 3.50 | 0.312 | 233,410 | 7.08 | 32,968 |
| 8. TS+ CP + single room + WSC | 622,687 | 16.95 | 0.661 |  |  | Dominated |
| 6. US + DNS | 694,808 | 0.79 | 0.022 | 132,583 | 2.72 | 48,744 |
| 12. US + DNS + WSC | 720,676 | 0.26 | 0.019 | 25,868 | 0.52 | 49,746 |
| 5. US + CP + single room | 785,495 | 16.85 | 0.605 |  |  | Dominated |
| 11. US + CP + single room + WSC | 798,201 | 15.77 | 0.622 |  |  | Dominated |

TS- targeted screening, US- universal screening, WSC- weekly screening

**Table 7. Impact of higher prevalence at admission (1% vs 0.1% in central analysis) combined with the lower identification of carriers by a risk-based screening (20% vs 50% in central analysis).**

| Strategy | Total cost/1000 admissions | Nb of CPE acquisitions/1000 admissions | Nb of CPE acquisitions/1000 admissions (central analysis) | Δ Cost/1000 admissions (€) | Δ Nb of CPE acquisitions/1000 admissions | ICER (€) |
| --- | --- | --- | --- | --- | --- | --- |
| Baseline | 39,045 | 6.41 | 0.929 |  |  | Dominated |
| 1. TS + CP | 54,067 | 5.50 | 0.776 |  |  | Dominated |
| 2. TS + CP + single room | 54,074 | 5.38 | 0.681 |  |  | Dominated |
| 3. TS + DNS | 72,315 | 4.11 | 0.335 | 33,270 | 2.30 | 14,465 |
| 8. TS+ CP + single room + WSC | 98,859 | 4.72 | 0.661 |  |  | Dominated |
| 7. TS+ CP + WSC | 100,663 | 4.82 | 0.777 |  |  | Dominated |
| 9. TS + DNS + WSC | 112,545 | 2.54 | 0.312 | 40,230 | 1.57 | 25,624 |
| 4. US + CP | 156,681 | 4.57 | 0.724 |  |  | Dominated |
| 5. US + CP + single room | 163,724 | 4.51 | 0.605 |  |  | Dominated |
| 11. US + CP + single room + WSC | 177,313 | 4.23 | 0.622 |  |  | Dominated |
| 10. US + CP+ WSC | 177,579 | 4.29 | 0.722 |  |  | Dominated |
| 6. US + DNS | 204,449 | 0.20 | 0.022 |  |  |  |
| 12. US + DNS + WSC | 206,705 | 0.09 | 0.019 | 94,160 | 2.45 | 38,433 |

TS- targeted screening, US- universal screening, WSC- weekly screening

**Table 8. Scenario with a CPE case identified at admission.**

| Strategy | Total cost/1000 admissions | Nb of CPE acquisitions/1000 admissions | Nb of CPE acquisitions/1000 admissions (central analysis) | Δ Cost/1000 admissions (€) | Δ Nb of CPE acquisitions/1000 admissions | ICER (€) |
| --- | --- | --- | --- | --- | --- | --- |
| Baseline | 32,962 | 1.624 | 0.929 |  |  |  |
| 2. TS + CP + single room | 37,619 | 0.901 | 0.681 | 4,657 | 0.72 | 6,468 |
| 1. TS + CP | 37,782 | 1.023 | 0.776 |  |  | Dominated |
| 8. TS+ CP + single room + WSC | 39,276 | 0.845 | 0.661 |  |  | Dominated |
| 7. TS+ CP + WSC | 39,662 | 0.968 | 0.777 |  |  | Dominated |
| 3. TS + DNS | 46,544 | 0.259 | 0.335 | 8,924 | 0.64 | 13,944 |
| 9. TS + DNS + WSC | 47,193 | 0.223 | 0.312 | 650 | 0.04 | 16 250 |
| 4. US + CP | 85,457 | 0.937 | 0.724 |  |  | Dominated |
| 5. US + CP + single room | 86,660 | 0.850 | 0.605 |  |  | Dominated |
| 11. US + CP + single room + WSC | 87,016 | 0.837 | 0.622 |  |  | Dominated |
| 10. US + CP+ WSC | 87,240 | 0.942 | 0.722 |  |  | Dominated |
| 6. US + DNS | 97,231 | 0.016 | 0.022 | 50,038 | 0.21 | 238,276 |
| 12. US + DNS + WSC | 97,688 | 0.015 | 0.019 | 457 | 0.001 | 457,000 |

TS- targeted screening, US- universal screening, WSC- weekly screening

**Table 9. Impact of higher cost of a hospital bed-day (1 000 € vs 500 € in central analysis).**

| Strategy | Total cost/1000 admissions | Nb of CPE acquisitions/1000 admissions | Total cost/1000 admissions (central analysis) | Δ Cost/1000 admissions (€) | Δ Nb of CPE acquisitions/1000 admissions | ICER (€) |
| --- | --- | --- | --- | --- | --- | --- |
| Baseline | 32,810 | 0.929 | 32,050 |  |  |  |
| 1. TS + CP | 41,366 | 0.776 | 37,304 |  |  | Dominated |
| 2. TS + CP + single room | 41,705 | 0.681 | 37,509 |  |  | Dominated |
| 8. TS+ CP + single room + WSC | 43,298 | 0.661 | 38,455 |  |  | Dominated |
| 7. TS+ CP + WSC | 43,461 | 0.777 | 38,560 |  |  | Dominated |
| 3. TS + DNS | 46,276 | 0.335 | 42,320 | 13,466 | 0.59 | 24,178 |
| 9. TS + DNS + WSC | 47,165 | 0.312 | 42,934 | 889 | 0.02 | 47,850 |
| 4. US + CP | 93,553 | 0.724 | 86,165 |  |  | Dominated |
| 5. US + CP + single room | 94,862 | 0.605 | 87,345 |  |  | Dominated |
| 11. US + CP + single room + WSC | 95,165 | 0.622 | 87,151 |  |  | Dominated |
| 10. US + CP+ WSC | 95,277 | 0.722 | 87,231 |  |  | Dominated |
| 6. US + DNS | 102,727 | 0.022 | 95,427 |  |  | Dominated |
| 12. US + DNS + WSC | 102,894 | 0.019 | 95,561 | 55,729 | 0.29 | 194,845 |

TS- targeted screening, US- universal screening, WSC- weekly screening

**Table 10. Scenario with the LOS of unidentified CPE cases = LOS of uncolonisad patients.**

| Strategy | Total cost/1000 admissions | Nb of CPE acquisitions/1000 admissions | Total cost/1000 admissions (central analysis) | Δ Cost/1000 admissions (€) | Δ Nb of CPE acquisitions/1000 admissions | ICER (€) |
| --- | --- | --- | --- | --- | --- | --- |
| Baseline | 32,202 | 0.465 | 32,050 |  |  |  |
| 1. TS + CP | 39,902 | 0.466 | 37,304 |  |  |  |
| 2. TS + CP + single room | 40,102 | 0.404 | 37,509 |  |  |  |
| 7. TS+ CP + WSC | 40,438 | 0.482 | 38,455 |  |  |  |
| 8. TS+ CP + single room + WSC | 40,649 | 0.406 | 38,560 |  |  |  |
| 3. TS + DNS | 45,326 | 0.136 | 42,320 | 13,124 | 0.330 | 39,770 |
| 9. TS + DNS + WSC | 45,341 | 0.140 | 42,934 |  |  |  |
| 4. US + CP | 91,213 | 0.538 | 86,165 |  |  |  |
| 10. US + CP+ WSC | 91,805 | 0.549 | 87,231 |  |  |  |
| 11. US + CP + single room + WSC | 92,197 | 0.487 | 87,151 |  |  |  |
| 5. US + CP + single room | 92,321 | 0.476 | 87,345 |  |  |  |
| 12. US + DNS + WSC | 100,203 | 0.011 | 95,427 | 54,877 | 0.120 | 457,308 |
| 6. US + DNS | 100,328 | 0.011 | 95,561 |  |  |  |

TS- targeted screening, US- universal screening, WSC- weekly screening

**Figure 1. Standard precautions (SP) compared with the targeted screening (TS) and contact precautions (CP) for different level of HH.**
